# An Inexpensive and Quick Method for Genotyping of HLA Variants Included in the Spanish Pharmacogenomic Portfolio of National Health System

**DOI:** 10.3390/ijms252011207

**Published:** 2024-10-18

**Authors:** Irene Taladriz-Sender, Gina Hernández-Osio, Paula Zapata-Cobo, Sara Salvador-Martín, Xandra García-González, Antonio Balas, María Sanjurjo-Sáez, Luis A. López-Fernández

**Affiliations:** 1 Servicio de Farmacia, Hospital General Universitario Gregorio Marañón, Instituto de Investigación Sanitaria Gregorio Marañón, 28007 Madrid, Spain; irene.taladriz@salud.madrid.org.com (I.T.-S.); gina.hernandez@iisgm.com (G.H.-O.); paula.zapata@iisgm.com (P.Z.-C.); sara.salvador@salud.madrid.org (S.S.-M.); xandra.garcia@salud.madrid.org (X.G.-G.); maria.sanjurjo@salud.madrid.org (M.S.-S.); 2 Histocompatibilidad Centro de Transfusión de Madrid, 28009 Madrid, Spain; abalas.trans@salud.madrid.org

**Keywords:** pharmacogenomics, pharmacogenetics, HLA-typing, genotyping

## Abstract

The possibility of using the same genotyping technology (TaqMan) for all the genetic tests included in the new Spanish pharmacogenomics portfolio should enable the application of a multigenotyping platform to obtain a whole pharmacogenomics profile. However, HLA-typing is usually performed with other technologies and needs to be adapted to TaqMan assays. Our aim was to establish a set of TaqMan assays for correct typing of HLA-A*31:01, HLA-B*15:02, HLA-B*57:01, and HLA-B*58:01. Therefore, we searched for and selected SNVs described in different populations as surrogate markers for these HLA alleles, designed TaqMan assays, and tested in a set of samples with known *HLA-A* and *HLA-B*. HLA-A*31:01 was correctly typed with a combination of rs1061235 and rs17179220 (PPV 100%, 95% CI 84.6–100-%; NPV 100%, 95% CI 96.5–100.0%), HLA-B*15:02 with rs10484555 (PPV 100%, 95% CI 69.2–100.0%; NPV 100%, 95% CI 96.8–100.0%) and rs144012689 (PPV 100%, 95% CI 69.2–100.0%; NPV 100%, 95% CI 96.8–100.0%), and HLA-B*57:01 with rs2395029 (PPV 99.5%, 95% CI 72.9–99.3%; NPV 99.5%, 95% CI 98.3–100.0%). HLA-B*58:01 was typed using two allele-specific TaqMan probes mixed with a *ß-Globin* reference and treated as a genotyping assay (PPV 100.0%, 95% CI 81.5–100.0%; NPV 100%, 95% CI 96.8–100.0%). In conclusion, we demonstrated a clinically useful way to type *HLA-A* and *HLA-B* alleles included in the Spanish pharmacogenomics portfolio using TaqMan assays.

## 1. Introduction

In June 2023, the Spanish National Health System updated the portfolio of genetic tests. For the first time, this update included a list of pharmacogenomic tests [1]. This portfolio contains 33 drug-gene pairs that should be available in all Spanish public centers if the patient meets the inclusion criteria. For this reason, many centers are including these genetic tests in their portfolio in an easy and inexpensive way. In our laboratory, we chose an OpenArray platform that works with TaqMan assays (Life Technologies, Carlsbad, CA, USA) to detect single-nucleotide variants (SNVs). 

This portfolio includes the following HLA alleles:

- HLA-A*31:01 for patients who are candidates for treatment with carbamazepine and at risk of severe adverse reactions (patients with Asian ancestry, with a personal or family history of severe skin toxicity associated with other drugs, or with previous severe adverse reactions after treatment with carbamazepine).
- HLA-B*15:02 for patients who are candidates for treatment with carbamazepine, phenytoin, or oxcarbazepine and at risk of severe adverse reactions (patients with Asian ancestry, with a personal or family history of severe skin toxicity associated with other drugs, or with previous severe adverse reactions after treatment with carbamazepine, phenytoin, or oxcarbazepine).
- HLA-B*58:01 for patients who are candidates for treatment with allopurinol and at risk of severe adverse reactions, especially in Asian and African populations.
- HLA-B*57:01 for patients who are candidates for treatment with abacavir.

Next-generation sequencing (NGS) procedures for HLA-typing are currently considered the gold standard for high-resolution allele assignment [2,3]. These techniques typically rely on short-read sequencing platforms, such as Illumina and Ion Torrent. However, short-read sequencing methods face challenges in resolving exon phasing for genes with long intronic regions, such as those found in HLA class II genes. The adoption of long-read sequencing technologies, such as PacBio and Oxford Nanopore, will provide complete phasing for all HLA genes, irrespective of intron length, greatly enhancing the accuracy and efficiency of HLA-typing [4]. However, this technology is still expensive and time-consuming. PCR and hybridization with sequence-specific oligonucleotide probes (PCR-SSOP) are also commonly applied in HLA-typing. Both techniques are useful only if the HLA/drug pair is to be identified. However, using a unique approach to obtain data for all the pharmacogenetic biomarkers included in the portfolio has multiple advantages in terms of cost-effectiveness and makes it possible to obtain a complete pharmacogenetic profile. This might be included in the patient’s clinical record for the rest of his/her life. For this purpose, we selected a multi-SNV approach based on TaqMan probes to obtain a full pharmacogenetic profile. However, the most accurate approaches for the determination of HLA cannot be used. The analyses to detect specific HLA alleles for genotyping of SNVs by real-time PCR using surrogate markers are usually based on reporter SNVs in linkage disequilibrium [5,6,7,8,9]. Of note is that most studies have been performed in Asian populations, and their usefulness in Caucasian populations is uncertain.

In this study, we analyzed the potential of several SNVs to correctly identify samples carrying HLA-B*57:01, HLA-B*15:02, HLA-B*58:01, and HLA-A*31:01 in a population from a Spanish hospital in order to incorporate them in a multigenotyping platform based on TaqMan assays. 

## 2. Results

### 2.1. HLA-A*31:01 Genotyping

Two SNVs were genotyped and compared to 125 samples with a known *HLA-A* type. The ability to genotype rs1061235 or rs17179220 for the detection of HLA-A*31:01 carriers in the population attending Spanish hospitals was good, with a negative predictive value (NPV) of 100%. However, both SNVs failed to correctly genotype the positive patients for HLA-A*31:01, with a positive predictive value (PPV) of 81.5 and 78.6%, respectively. PPV, NPV, specificity, and sensitivity were 100% when patients carried both polymorphisms (Table 1, Table 2 and Table 3).

There was not a complete correspondence between the heterozygous status for these SNVs and carrying one or two HLA-A*31:01 alleles. Most individuals were heterozygous for the variants analyzed and carried only one HLA-A*31:01 allele. However, three samples were homozygous for the SNV rs1061235 and carried HLA-A*31:01 allele, and two were heterozygous for the SNV rs1061235 and carried two HLA-A*31:01 alleles. Similarly, two samples were homozygous for the SNV rs17179220 and carried HLA-A*31:01.

### 2.2. HLA-B*15:02 Genotyping

Two SNVs were genotyped and compared to 125 known samples for the HLA-B*15:02 allele type. Since SNV rs144012689 contains another genetic variant with two close nucleotides, two TaqMan assays were tested, as previously described [10]. The usefulness of these three TaqMan assays in two SNVs was excellent. The rs10484555 was useful for detecting HLA-B*15:02 carriers in the population attending Spanish hospitals (Table 4). The assays in SNV rs10484555 were validated with 123 of the 125 samples included; the result was indeterminate in two samples. The SNV rs144012689 was also valid for the correct classification of HLA-B*15:02 carriers in our population (Table 5). 

There was not a complete correspondence between the heterozygous status for these SNVs and carrying one or two HLA-B*15:02 alleles. Most individuals were heterozygous for the variants analyzed and carried only one HLA-B*15:02 allele. However, two samples were homozygous for the SNV rs144012689 or rs10484555 and carried one HLA-B*15:02 allele. Interestingly, only one sample carried two HLA-B*15:02 alleles, and all assays classified it as homozygous for the variant.

### 2.3. HLA-B*57:01 Genotyping

For validation of HLA-B*57:01, we used a total of 233 samples with a known HLA-B*57:01 type by applying different methods, namely, Luminex xMAP technology with reverse sequence-specific oligonucleotide (*n* = 66) [11] and allele-specific PCR (AS-PCR) with melting curve analysis (*n* = 167) [12]. All these samples were genotyped for rs2395029. The results of the comparison are shown in Table 6. Concordance between Luminex and AS-PCR was 100% (*n* = 66). 

This comparison reveals a PPV of 95.0%, NPV of 100.0%, specificity of 99.5%, and sensitivity of 100.0%. Only one discrepancy was observed. This sample was sequenced with HLA-B*57:01, yielding a negative result. 

The typing method for most of the samples for HLA-B*57:01 did not allow us to know how many HLA-B*57:02 alleles they carry.

### 2.4. HLA-B*58:01 Genotyping

Three SNVs previously associated with the HLA-B*58:01 allele were genotyped and compared to 132 known samples for the HLA-B*58:01 allele type. The usefulness of these three SNVs was very low. Genotyping of rs9263726 was not useful for detecting HLA-B*58:01 carriers in the population seen in Spanish hospitals (Table 7). There were 35 false positives and eight false negatives in 132 samples, with a very low sensitivity (52.9%) and a specificity of 69.6%. 

Similar results were observed for rs9262570 and rs9469003 (Table 8 and Table 9), with sensitivities between 31.3% and 94.4%. The rs9469003 variant was very sensitive (94.4%) but had low sensitivity (70.2%).

**Figure 1:**
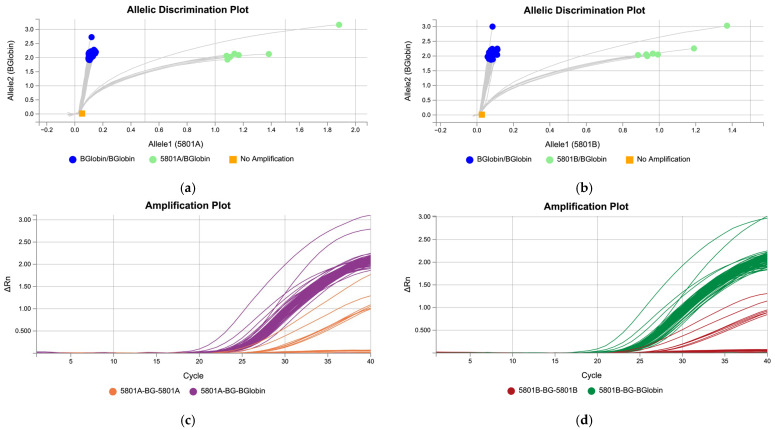
Allelic discrimination and amplification plots for specifically designed HLA-B*58:01 assays. Carriers of HLA-B*5801 were perfectly clustered. The figure shows the results of the following: (**a**) the allelic discrimination plot assay for 5801A (green, HLA-B*5801 positive and *ß-Globin* positive; blue, HLA-B*5801 negative and *ß-Globin* positive); (**b**) the allelic discrimination plot assay for 5801B (green, HLA-B*5801 positive and *ß-Globin* positive; blue, HLA-B*5801 negative and *ß-Globin* positive); (**c**) the amplification plot assay for 5801A (purple, *ß-Globin*, orange, HLA-B*5801); and (**d**) the amplification plot assay for 5801B (green, *ß-Globin*; red, HLA-B*5801).

Since the SNVs analyzed were not useful for detecting HLA-B*58:01 carriers and we needed to use a TaqMan assay, we designed two “false” TaqMan assays using the sequences provided by Zhang et al., who detected HLA-B*58:01 with an allele-specific PCR and TaqMan probes [13]. These assays were developed to work with standard reagents, temperatures, and times for a genotyping TaqMan assay in order to be included in an OpenArray customized chip. Both assays contained two oligonucleotides specific for HLA-B*58:01 and a 5801-specific probe (5801A or 5801B), two oligonucleotides specific for *ß-Globin*, and a specific probe for *ß-Globin*. The amplification was then analyzed using allelic discrimination plots, as is usual practice in genotyping TaqMan assays (Figure 1).

The concordance of genotyping using both assays was 99.24%. Only one sample, which had an HLA-B*58:06 allele using Luminex technology, was genotyped as HLA-B*58:01 with the new method. After sequencing as described by Lazaro et al. [14], the sample was re-classified as HLA-B*58:01. Thus, concordance was finally shown to be absolute (Table 10).

## 3. Discussion

The approval of the Spanish pharmacogenomic portfolio provides an excellent opportunity to include a pharmacogenetic profile in patients’ clinical records. We selected a multigenotyping platform based on a TaqMan assay as a cost-effective method to obtain a whole pharmacogenetic profile. This profile should contain several HLA alleles associated with severe adverse reactions to drugs such as allopurinol, carbamazepine, and abacavir [15,16]. Our study validates the use of TaqMan assays to type HLA-A*31:01, HLA-B*15:02, HLA-B*57:01, and HLA-B*58:01 so that they can be included in the multigenotyping platform.

The HLA-B*57:01 genotype inferred using the G allele of rs2395029 is accepted and commonly used in clinical practice [9]. Zubiaur et al., analyzed 226 samples (49 positive and 177 negative for HLA-B*57:01) and did not find any discrepancy between the G allele of rs2395029 and HLA-B*57:01. In an older study with 119 samples, the result was identical [17]. In our study, we analyzed 233 samples, and, for the first time in the literature, we found one discrepancy. One sample was positive for the G allele of rs2395029 and negative for HLA-B*57:01. This finding supports the inclusion of a warning in the clinical pharmacogenetics reports for HLA-B*57:01 inferred by genotype rs2395929, indicating an incorrect identification. 

Correct determination of HLA-B*57:01 goes beyond avoiding a hypersensitivity reaction. It was recently shown that the HLA-B*57:01 allele corresponded to a very large MHC haploblock, likely explaining its massive effect on elite control of HIV-1 [18]. Although rs2395029 genotyping is not perfect for HLA-B*57:01 testing, it is the quickest and most inexpensive way to perform this pharmacogenetic test, enabling it to be included in a multi-SNV determination, together with other important pharmacogenetic variants included in the Spanish pharmacogenomics portfolio.

The results for HLA-B*15:02 showed high sensitivity and specificity (NPV 100%, PPV 100%, 100% sensitivity, and 100% specificity) for both SNVs analyzed, namely, rs144012689 and rs10484555. HLA-B*15:02 allele-typing is thought to be inferred by several surrogate SNV markers, namely, rs31451122, rs3909184, rs2844682, rs144012689, and rs10484555 [10,19,20,21,22]. The first three were ruled out in this study owing to the low sensitivity and specificity found in other studies or because linkage was insufficiently demonstrated [23]. The last two SNVs have been tested in mixed populations (e.g., Asians, African Americans, Caucasians, and Hispanics) with 100% sensitivity and >98.0% specificity [10,19]. This was the reason they were selected for our study. Since both SNVs seem to correctly identify HLA-B*15:02 in patients seen in Spanish hospitals, they should both be included (three TaqMan assays) in a multigenotyping system and checked for any discrepancy between them to explore which is the most useful when thousands of samples are genotyped.

HLA-A*31:01 alleles showed the combination of rs1061235 and rs17179220 to be useful for HLA-A*31:01–inferred typing in clinical practice. While both SNVs have been proposed for inferred allele-typing in different populations, their usefulness in Spanish populations had not previously been tested [10], and although both SNVs showed good sensitivity and specificity, errors were detected in the correct classification for rs1061235 [23,24]. In our study, we observed that specificity was not 100% for either of the two SNVs. However, the combination of both SNVs was able to type 100% of samples for HLA-A*31:01. For this reason, both SNVs should be included in the multigenotyping test for correct detection of HLA-A*31:01 carriers. 

In clinical practice, we genotyped HLA-B*58:01 using allele-specific real-time PCR as described elsewhere [25]. However, this methodology cannot be included in a multigenotyping system using TaqMan probes. A test with a unique TaqMan assay has been described for HLA-B*57:01 and HLA-B*58:01 screening [26]. The genotyping of HLA-B*58:01 using a single SNV has been described for Asian populations (rs9263726, rs2734583, and rs3099844), with limited results [27]. Despite many efforts to find an SNV as a surrogate marker for HLA-B*58:01, none has been validated, especially in Caucasian populations and not in Spain [13,23,28,29]. For instance, HLA-B*58:01 was tagged by the SNV rs9262570 at 100% sensitivity and >95% specificity in a Chinese Han population [29]. However, in our population, the sensitivity of this SNV for typing of HLA-B*58:01 was 26.3% and the specificity was 90.3%. The results are similar to those of the other SNVs tested for this HLA-B allele. These values are too low to be used in clinical practice. In this study, and adapting from a previous work [13], we developed two allele-specific TaqMan probes that work as genotyping TaqMan assays. This adaptation enabled us to include these assays in a multigenotyping platform, together with real genotyping TaqMan assays, such as OpenArray. The PPV, NPP, specificity, and sensitivity enable it to be used in clinical practice. The evidence that it is possible to mix allele-specific assays in the same platform with genotyping assays makes it possible to develop new allele-specific assays for all the valuable HLA alleles in pharmacogenetic analysis.

There is not a perfect correspondence between being heterozygous or homozygous for the variants analyzed and carrying one or two HLA-A*31:01 or HLA-B*15:02 alleles. This was also observed with the SNV rs2097432 and HLA-DQA1*05 [30]. This suggests that the selected surrogate markers are useful for identifying the presence of a specific HLA but not for determining the number of them.

TaqMan genotyping is an inexpensive technology for pharmacogenetic testing [31]. Moreover, using multiprobe systems, such as OpenArray (ThermoFisher, Waltham, MA, USA), makes it possible to obtain a complete profile of pharmacogenetic information, including HLA genes, at a reduced cost. This information will be stored and consulted when needed, thus avoiding new pharmacogenetic tests for the same patients in the future. It will allow us to reformulate cost-effective studies. Thus, Zhou et al. estimated the global frequencies of HLA-B*57:01, HLA-B*58:01, HLA-B*15:02, and HLA-A*31:01 and assessed their implications for the cost-effectiveness of preemptive pharmacogenetic testing [32]. Since the cost estimated by Zhou et al., for genotyping of HLAs was between $40 and $141, a preemptive approach with a complete pharmacogenetic profile containing relevant HLA alleles will be more useful and cost-effective. 

A limitation of the study was the unavailability of ethnicity data for the samples tested. However, most patients seen in our hospital are Caucasian. Therefore, while the conclusions may be applicable to other European countries, caution should be exercised when considering patients of other ethnicities. The advantage of using an allele-specific TaqMan assay for HLA-B*58:01 is that it is not ethnicity-dependent.

In conclusion, for patients seen in the Spanish National Health System, we demonstrated the clinical usefulness of the SNVs rs1061235 and rs17179220 for allele-typing of HLA-A*31:01, rs144012689 and rs10484555 for HLA-B*15:02, rs2395029 for HLA-B*57:01, and the optimized allele-specific 5801A and 5801B for HLA-B*58:01. In addition, we showed that allele-specific TaqMan probes might also be used as genotyping assays, thus avoiding the need for surrogate markers.

## 4. Materials and Methods

### 4.1. Control Samples and TaqMan Genotyping

Samples with known HLA from the Pharmacogenetics Laboratory of Gregorio Marañón Hospital and Blood Transfusion Center, Madrid, Spain, were used for comparison with the SNVs studied. *HLA-A* and *HLA-B* were previously analyzed in these samples using Luminex technology combined with PCR amplification based on sequence-specific oligonucleotides (Lifecodes, Stamford, CT, USA) [11] and Sanger sequencing at the Genomics Unit of Gregorio Marañón Hospital. SnapGene version 5.3 or AlleleSEQR^®^HLA (Utrecht, The Netherlands) was used for sequence analysis. Additionally, HLA genes from certain control samples were analyzed using NGS procedures (GenDX, Utrecht, The Netherlands) and a MiSeq platform (Illumina, San Diego, CA, USA). NGS data were analyzed using the NGSengine Software 2.30.1 (GenDX, Utrecht, The Netherlands) and the 3.55 version of the IPD-IMGT/HLA Database (https://www.ebi.ac.uk/ipd/imgt/hla/; accessed on 1 August 2024). For HLA-B*57:01, we increased the sample size using an in-house AS-PCR and melting curve [12]. A total of 11 TaqMan assays (Life Technologies, Carlsbad, CA, USA) were tested to cover HLA-B*57:01, HLA-B*15:02, HLA-B*58:01, and HLA-A*31:01 (Table 11).

TaqMan assays (Life Technologies, Carlsbad, CA, USA) were used for genotyping as follows: PCR was performed by mixing 2.5 µL of TaqPath ProAmp Master Mix 2X (Life Technologies, Carlsbad, CA, USA), 0.250 µL of TaqMan assay 20X or 0.125 µL of TaqMan assay 40X, and 1 µL of DNA (10 ng/µL) in a final volume of 5 µL, with the following steps: 60 °C for 30 s; 95 °C for 5 min; 40 cycles of 95 °C for 15 s, 60 °C for 1 min; and a final extension at 60 °C for 30 s. Fluorescence was measured at the start and end of the process and at every cycle after extension.

Sequencing was performed on the discrepant sample of HLA-B*5701 and HLA-B*58:01, as described by Lazaro et al. [14].

### 4.2. HLA-B*58:01 Allele-Specific TaqMan Assay

The two new HLA-B*58:01 TaqMan assays, 5801A and 5801B, were designed using seven oligonucleotides described elsewhere [13] by mixing oligonucleotides for an HLA-B*58:01–specific amplification (VIC) and a *ß-Globin* amplification (FAM). Concentrations of probes in the assay, PCR conditions, and reagents were optimized (Table 12).

PCR was performed by mixing 2.5 µL of TaqPath ProAmp Master Mix 2X, 0.250 µL of TaqMan assay 20X, and 1 µL of DNA (10 ng/µL) in a final volume of 5 µL, with the following steps: 60 °C for 30 s; 95 °C for 5 min; 40 cycles of 95 °C for 15 s, 60 °C for 1 min; and a final extension at 60 °C for 30 s. Fluorescence was measured at the start and end of the process and at every cycle after extension.

### 4.3. Statistical Analysis

The PPV, NPV, specificity, and sensitivity were calculated as follows: (a) sensitivity = (true positives)/(true positives + false negatives), (b) specificity = (true negatives)/(true negatives + false positives); (c) PPV = (true positives)/(true positives + false positives); and (d) NPV = (true negatives)/(true negatives + false negatives) [33]. The 95% confidence interval (CI) was calculated as described by Mercaldo and Zhou [34].

## 5. Conclusions

The combination of six TaqMan assays makes it possible to type the four HLA alleles included in the Spanish pharmacogenomic testing portfolio in clinical practice. The assays described are useful for allele-typing of HLA-A*31:01, HLA-B*15:02, HLA-B*57:01, and HLA-B*58:01 in patients seen in the Spanish National Health System. 

## Figures and Tables

**Table 1:** Concordance between validated HLA-A*31:01 genotyping methods and rs1061235 genotyping.

	rs1061235-Positive	rs1061235-Negative
Real positive HLA-A*31:01	22	0
Real negative HLA-A*31:01	5	98
	**Value**	**95% CI**
Sensitivity	100.0%	84.6% to 100.0%
Specificity	95.1%	89.0% to 98.4%
Positive predictive value	81.5%	65.2% to 91.2%
Negative predictive value	100.0%	96.3% to 100.0%

**Table 2:** Concordance between validated HLA-A*31:01 genotyping methods and rs17179220 genotyping.

	rs17179220-Positive	rs17179220-Negative
Real positive HLA-A*31:01	22	0
Real negative HLA-A*31:01	6	97
	**Value**	**95% CI**
Sensitivity	100.0%	84.6% to 100.0%
Specificity	94.2%	87.8% to 97.8%
Positive predictive value	78.6%	62.8% to 88.8%
Negative predictive value	100.0%	96.3% to 100.0%

**Table 3:** Concordance between validated HLA-A*31:01 genotyping methods and combined rs1061235/rs17179220 genotyping.

	rs1061235- and rs17179220-Positive	rs1061235 and rs17179220-Negative
Real positive HLA-A*31:01	22	0
Real negative HLA-A*31:01	0	103
	**Value**	**95% CI**
Sensitivity	100.0%	84.6% to 100.0%
Specificity	100.0%	96.5% to 100.0%
Positive predictive value	100.0%	84.6% to 100.0%
Negative predictive value	100.0%	96.5% to 100.0%

**Table 4:** Concordance between validated HLA-B*15:02 genotyping methods and rs10484555 genotyping.

	rs10484555-Positive	rs10484555-Negative
Real positive HLA-B*15:02	10	0
Real negative HLA-B*15:02	0	113
	**Value**	**95% CI**
Sensitivity	100.0%	69.2% to 100.0%
Specificity	100.0%	96.8% to 100.0%
Positive predictive value	100.0%	69.2% to 100.0%
Negative predictive value	100.0%	96.8% to 100.0%

**Table 5:** Concordance between validated HLA-B*15:02 genotyping methods and rs144012689 genotyping.

	rs144012689-Positive ^1^	rs144012689-Negative
Real positive HLA-B*15:02	10	0
Real negative HLA-B*15:02	0	115
	**Value**	**95% CI**
Sensitivity	100.0%	69.2% to 100.0%
Specificity	100.0%	96.8% to 100.0%
Positive predictive value	100.0%	69.2% to 100.0%
Negative predictive value	100.0%	96.8% to 100.0%

^1^ Samples positive for either of the two assays were considered positive.

**Table 6:** Concordance between validated HLA-B*57:01 genotyping methods and rs2395029 (HCP5) genotyping.

	rs2395029 (*HCP5*)-Positive	rs2395029 (*HCP5*)-Negative
Real positive HLA-B*57:01	19	0
Real negative HLA-B*57:01	1	213
	**Value**	**95% CI**
Sensitivity	100.0%	82.3% to 100.0%
Specificity	99.5%	97.4% to 99.9%
Positive predictive value	95.0%	72.9% to 99.3%
Negative predictive value	100.0%	98.3% to 100.0%

**Table 7:** Concordance between validated HLA-B*58:01 genotyping methods and rs9263726 genotyping.

	rs9263726-Positive	rs9263726-Negative
Real positive HLA-B*58:01	9	8
Real negative HLA-B*58:01	35	80
	**Value**	**95% CI**
Sensitivity	52.9%	27.8% to 77.0%
Specificity	69.6%	60.3% to 77.8%
Positive predictive value	20.5%	13.9% to 30.3%
Negative predictive value	90.9%	85.6% to 94.4%

**Table 8:** Concordance between validated HLA-B*58:01 genotyping and rs9262570 genotyping methods.

	rs9262570-Positive	rs9262570-Negative
Real positive HLA-B*58:01	5	11
Real negative HLA-B*58:01	14	102
	**Value**	**95% CI**
Sensitivity	31.3%	11.0% to 58.7%
Specificity	87.9%	80.6% to 93.2%
Positive predictive value	26.3%	12.9% to 46.2%
Negative predictive value	90.3%	86.9% to 92.8%

**Table 9:** Concordance between validated HLA-B*58:01 genotyping and rs9469003 genotyping methods.

	rs9469003-Positive	rs9469003-Negative
Real positive HLA-B*58:01	17	1
Real negative HLA-B*58:01	34	80
	**Value**	**95% CI**
Sensitivity	94.4%	72.7% to 99.9%
Specificity	70.2%	60.9% to 78.4%
Positive predictive value	33.3%	27.0% to 40.4%
Negative predictive value	98.8%	92.2% to 99.8%

**Table 10:** Concordance between validated HLA-B*58:01 genotyping methods and HLA-B*58:01–specific TaqMan probe genotyping.

	HLA-B*58:01-Specific TaqMan Positive	HLA-B*58:01-Specific TaqMan Negative
Real positive HLA-B*58:01	18	0
Real negative HLA-B*58:01	0	114
	**Value**	**95% CI**
Sensitivity	100.0%	81.5% to 100.0%
Specificity	100.0%	96.8% to 100.0%
Positive predictive value	100.0%	81.5% to 100.0%
Negative predictive value	100.0%	96.8% to 100.0%

**Table 11:** Single-nucleotide variants (SNVs) analyzed for HLA-inferred genotypes, and TaqMan assays analyzed.

Associated HLA	dbSNP ID	TaqMan ID	Reference
HLA-B*57:01	rs2395029	C__16222070_10	Zubiaur et al. [9]
HLA-B*15:02	rs10484555	ANEP7CD	Buchner et al. [10]
HLA-B*15:02	rs144012689	AN33NRP	Buchner et al. [10]
HLA-B*15:02	rs144012689	AN49HCM	Buchner et al. [10]
HLA-A*31:01	rs17179220	C__33415939_10	Buchner et al. [10]
HLA-A*31:01	rs1061235	ANKCPPX	Buchner et al. [10]
HLA-B*58:01	rs9262570	C__29757466_10	Liu et al. [29]
HLA-B*58:01	rs9263726	ANDKDRF	Maekawa et al. [28]
HLA-B*58:01	rs9469003	C_30505354_31	He et al. [23]
HLA-B*58:01	Allele-specific	5801A	Zhang et al. [13]
HLA-B*58:01	Allele-specific	5801B	Zhang et al. [13]

**Table 12:** Oligonucleotides used for HLA-B*58:01-specific TaqMan assays.

Oligo Name	Sequence	Concentration (20×)	TaqMan Assay
*ß-Globin*-F	AGTCAGGGCAGAGCCATCTA	10.8 µM	5801A and 5801B
*ß-Globin*-R	TTAGGGTTGCCCATAACAGC	10.8 µM	5801A and 5801B
*ß-Globin*	6FAM-AGTCTGCCGTTACTGCCCTGTGG-MGB	2.4 µM	5801A and 5801B
5801-AS-F	GGGCCGGAGTATTGGGATG	18 µM	5801A and 5801B
5801-AS-R	GCCATACATCCTCTGGATGA	18 µM	5801A and 5801B
5801-A	VIC-ACCGAGAGAACCTGCGGATCGCGCTCC-QSY	4 µM	5801A
5801-B	VIC-TCCGAGATCCGCCTCCCTGAGGCC-QSY	4 µM	5801B

AS, allele-specific.

## Data Availability

The original data presented in this study are openly available in “Repositorio de la Consejería de Sanidad de la Comunidad de Madrid” at https://hdl.handle.net/20.500.12530/87952 (accessed on 1 August 2024).
